# Expression of Myeloperoxidase in Patient-Derived Endothelial Colony-Forming Cells—Associations with Coronary Artery Disease and Mitochondrial Function

**DOI:** 10.3390/biom14101308

**Published:** 2024-10-16

**Authors:** Weiqian Eugene Lee, Elijah Genetzakis, Giannie Barsha, Joshua Vescovi, Carmen Mifsud, Stephen T. Vernon, Tung Viet Nguyen, Michael P. Gray, Stuart M. Grieve, Gemma A. Figtree

**Affiliations:** 1 Kolling Institute, 10 Westbourne Street, St Leonards, Sydney, NSW 2064, Australia; 2 The Victorian Heart Institute and Biomedicine Discovery Institute, Monash University, Wellington Road, Clayton, Melbourne, VIC 3800, Australia; 3 Charles Perkins Centre, Johns Hopkins Drive, Camperdown, Sydney, NSW 2050, Australia

**Keywords:** myeloperoxidase, endothelial dysfunction, endothelial colony forming cells, coronary artery disease, discovery

## Abstract

Background and Aims: Myeloperoxidase (MPO) plays a critical role in the innate immune response and has been suggested to be a surrogate marker of oxidative stress and inflammation, with elevated levels implicated in cardiovascular diseases, such as atherosclerosis and heart failure, as well as in conditions like rheumatoid arthritis and cancer. While MPO is well-known in leukocytes, its expression and function in human endothelial cells remain unclear. This study investigates MPO expression in patient-derived endothelial colony-forming cells (ECFCs) and its potential association with CAD and mitochondrial function. Methods: ECFCs were cultured from the peripheral blood of 93 BioHEART-CT patients. MPO expression and associated functions were examined using qRT-PCR, immunochemistry, flow cytometry, and MPO activity assays. CAD presence was defined using CT coronary angiography (CACS > 0). Results: We report MPO presence in patient-derived ECFCs for the first time. MPO protein expression occurred in 70.7% of samples (*n* = 41) which had nuclear co-localisation, an atypical observation given its conventional localisation in the granules of neutrophils and monocytes. This suggests potential alternative roles for MPO in nuclear processes. MPO mRNA expression was detected in 66.23% of samples (*n* = 77). CAD patients had a lower proportion of MPO-positive ECFCs compared to non-CAD controls (57.45% vs. 80%, *p* = 0.04), a difference that persisted in the statin-naïve sub-cohort (53.85% vs. 84.62%, *p* = 0.02). Non-CAD patients with MPO expression showed upregulated mitochondrial-antioxidant genes (*AIFM2*, *TXNRD1*, *CAT*, *PRDX3*, *PRDX6*). In contrast, CAD patients with MPO gene expression had heightened mROS production and mitochondrial mass and decreased mitochondrial function compared to that of CAD patients without MPO gene expression. Conclusions: MPO is present in the nucleus of ECFCs. In non-CAD ECFCs, MPO expression is linked to upregulated mitochondrial-antioxidant genes, whereas in CAD ECFCs, it is associated with greater mitochondrial dysfunction.

## 1. Introduction

Coronary artery disease (CAD) remains a leading cause of morbidity and mortality worldwide [1]. Despite advancements in our knowledge of the pathophysiology of CAD, the underlying molecular mechanisms are complex and poorly understood. Recent clinical evidence from the Canakinumab Anti-inflammatory Thrombosis Outcome Study (CANTOS) demonstrated the independent benefit of interleukin-1 beta (IL-1β) in a cohort with established residual risk indicated by elevated C-reactive protein (CRP) levels [2]. Still, the determinants of susceptibility to inflammation in CAD remain elusive.

Myeloperoxidase (MPO) is a haem enzyme primarily derived from leucocytes and has been well-associated with neutrophils and monocytes [3,4]. In classical immunological disorders, MPO is critical in host defence against pathogens like fungi and bacteria [5]. Through the catalysis of H_2_O_2_ and Cl^−^, which generates HOCl^−^, MPO exerts bactericidal effects against phagocytosed pathogens [6,7,8,9]. Murine studies have also shown that MPO-deficient animals are more susceptible to infections and demonstrate an exaggerated inflammatory response [5]. However, the non-specific nature of HOCl^−^ has been linked to detrimental outcomes, promoting mitochondrial dysfunction and compromising endothelium-dependent relaxation by diminishing nitrous oxide (NO) bioavailability in endothelial cells [10]. As such, the role of MPO as a pathogenic mediator of endothelial dysfunction is an important question that needs to be investigated.

Substantial ex vivo and preclinical evidence has linked MPO expression with atherosclerotic plaque destabilization [11,12]. Treatment with an MPO inhibitor, AZM198, significantly improved femoral artery relaxation in response to acetylcholine (ACh) [13]. However, it remains unknown whether MPO released from leukocytes promotes endothelial dysfunction or if endothelial cells act as their own reservoir of MPO. Recent studies have identified endogenous MPO expression within human umbilical vein, aortic and endocardial endothelial cell lines, revealing a potential dual impact on vascular function, with both protective and damaging effects reported [14,15,16]. While exogenous and circulating MPO has been extensively studied and implicated in the progression of atherosclerosis, the expression of endogenous MPO within endothelial cells and its association with coronary disease remains unclear. We recently demonstrated that CAD patients’ endothelial colony-forming cells (ECFCs) show increased production of mitochondrial reactive oxygen species (mROS) compared to those from non-CAD patients [17,18,19]. As such, ECFCs may provide an opportunity to dissect the relationship, given they have been found to retain the molecular and functional imprints of a patient’s CAD state. Thus, they are a crucial model for human patient-derived drug and biomarker discovery.

In this study, we evaluate whether patient-derived ECFCs demonstrate their own reservoir of MPO that may contribute to mitochondrially-associated endothelial dysfunction and CAD in a unique CTCA-characterised cohort and biobank.

## 2. Materials and Methods

### 2.1. PBMC Isolation, ECFC Growth and Cell Culture

Peripheral blood samples from BioHEART-CT patients were collected immediately after the insertion of the peripheral venous cannula required for CCTA. Blood tubes with lithium heparin anticoagulant were utilised for PBMC isolation. Following collection, lithium heparin tube(s) were stored at room temperature until processing. Within four hours of collection, PBMCs were isolated using a standard Ficoll preparation method [20]. The PBMCs were then plated onto T-25 flasks coated with 0.1% gelatine (ThermoFisher Scientific, Waltham, MA, USA) at a density of 2.5 × 10^4^ cells/cm^2^. Endothelial cell growth medium (EGM-2) (Lonza, Basel, Switzerland), supplemented with the EGM-2 SingleQuot Kit and 2% FBS, was used as the culture medium. The flasks containing the plated PBMCs were cultured at 37 °C with 5% CO_2_ for up to 21 days, with regular monitoring of spontaneous ECFC growth. Spontaneous growth occurred in 21.5% of patients, in a manner that was slightly higher in those with CAD [18]. As the ECFCs matured, cell lines were expanded into T-75 flasks and 10 cm^2^ dishes for subsequent analyses. Upon reaching passage 4, the successfully matured ECFCs were cryopreserved in a solution of 10% dimethyl sulfoxide (DMSO) and 90% FBS. The cryopreserved ECFCs were stored in LN_2_ at −80 °C for long-term preservation and future use in downstream experiments and analyses. ECFCs used in this study were between passages 4 and 6.

Human umbilical vein endothelial cells (HUVECs) were purchased from ATCC and cultured in EGM-2 medium (ATCC^®^ PCS-100-013™) supplemented with 2% FBS and the EGM-2 SingleQuot Kit. HUVECs were used between passages 4–6.

### 2.2. Study Population and Recruitment

Blood samples were obtained from the BioHEART-CT cohort (Australia New Zealand Clinical Trials Registry ANZTR12618001322224, Camperdown, Australia), a multi-centre, prospective cohort study of patients referred for coronary CT angiography (CCTA) [21]. Adults with previously diagnosed or suspected CAD were invited to provide written informed consent for this study. Clinical data was collected via facilitated interview at the time of recruitment and included demographics, anthropometrics, past medical history, family history, medications, occupational and exposure history and indication for CCTA. Participants with prior history of myocardial infarction, percutaneous coronary intervention or coronary artery bypass graft were excluded. Ninety-three participants meeting these criteria, where ECFCs were available, were included in the cell function analysis. Patients included in this study were recruited between 2016 and 2022. This study was conducted according to the guidelines of the 1975 Declaration of Helsinki and approved by the Northern Sydney Local Health District Human Research Ethics Committee (2019/ETH08376).

### 2.3. Imaging Analysis

CCTAs were performed using a 256-slice CT scanner, in which standard clinical protocols was used for the creation of reconstructions as described previously [18,19,21,22]. CAD was considered CACS > 0, as per the Australian clinical recommendations for CACS [23,24].

### 2.4. Western Blot

Patient-derived ECFCs were lysed in ice-cold RIPA lysis buffer supplemented with PhosSTOP and cOmplete ULTRA (Sigma Aldrich, St Louis, MO, USA). Protein concentration was measured with a Pierce^TM^ BCA Protein assay kit (Thermo Fisher Scientific, Sydney, NSW, Australia). Equal protein amounts were prepared in sample buffer and denatured (70 °C, 10 min). Samples were loaded and resolved by SDS-PAGE. Protein was transferred onto a Trans-Blot Turbo 0.2 μm PVDF membrane (BioRad, South Granville, NSW, Australia) and blocked with Pierce^TM^ Protein-Free Blocking Buffer (ThermoFisher Scientific, Sydney, NSW, Australia) (room temperature, 1 h). The membrane was probed with polyclonal anti-MPO (1:500, Cat no: 22225-1-AP, Proteintech, San Diego, CA, USA) and monoclonal anti-β actin (1:5000, Cat no: 60008-1-Ig, Proteintech) antibody (overnight, 4 °C). Secondary fluorescent antibodies specific to primary antibodies were then used (donkey anti-rabbit IRDye 680LT, LCR-926-32210 and goat anti-mouse 800CW, LCR-926-68023; Millenium Science, Mulgrave, Australia). Membranes were imaged using the ChemiDoc^TM^ MP (BioRad, South Granville, NSW, Australia) and analysed with Image Lab software (BioRad, Hercules, CA, USA, Version 6.1). PBMCs were used as positive control and HUVECs were used as negative control.

### 2.5. Subcellular Protein Fractionation

Subcellular protein fractionation for patient-derived ECFCs was performed as per manufacturer’s instructions (Cat no: 78840, Thermo Fisher Scientific, Waltham, MA, USA). Briefly, ECFCs were lifted and centrifuged (500× *g*, 5 min). The supernatant was removed and resuspended in ice-cold PBS and centrifuged (500× *g*, 3 min). The supernatant was removed and subcellular protein fractionation was performed in a step-wise manner to obtain cytoplasmic, membrane, soluble nuclear and chromatin-bound nuclear extracts, respectively.

Firstly, 100 μL ice-cold cytoplasmic extraction buffer (CEB) containing protease inhibitor was added to the cell pellet and incubated for 10 min at 4 °C with gentle mixing. Subsequently, the mixture was centrifuged at 500× *g* for 5 min where afterwards the cytoplasmic extract (supernatant) was transferred to a new pre-chilled Eppendorf on ice. A 100 μL volume of ice-cold membrane extraction buffer (MEB) containing protease inhibitor was added, vortexed (5 s) and incubated for 10 min at 4 °C with gentle mixing. The mixture was centrifuged at 3000× *g* for 5 min and the membrane extract (supernatant) was transferred to a new pre-chilled Eppendorf on ice. A 50 μL volume of ice-cold nuclear extraction buffer (NEB) containing protease inhibitor was added, vortexed (15 s) and incubated for 30 min at 4 °C with gentle mixing. The mixture was then vortexed at 5000× *g* for 5 min and nuclear extract (supernatant) to a new pre-chilled Eppendorf on ice. Chromatin-bound extraction buffer was prepared as per manufacturer’s instructions and added to the pellet and vortexed (15 s) before being incubated at 37 °C for 5 min. The mixture was then vortexed again (15 s) and centrifuged at 16,000× *g* for 5 min. The chromatin-bound nuclear extract was transferred to a new pre-chilled Eppendorf on ice. Fractions were either maintained on ice for same-day downstream analysis or stored at −80 °C.

### 2.6. Immunocytochemistry

Patient-derived ECFCs were seeded into 48-well plates overnight. The medium was removed and cells were then washed once with PBS. The cells were fixed with 1% formalin (10 min, room temperature). The cells were PBS-washed twice and then permeabilised with Triton (room temperature, 30 min). The cells were PBS-washed twice and blocked with 2.5% BSA (room temperature, 20 min). Monoclonal anti-MPO (1:50 dilution) was added and incubated overnight (4 °C). The cells were then washed thrice with PBS and 2° antibody (anti-rabbit, 1:2000 dilution) was added (45 min, room temperature) and protected from light. Afterwards, the cells were washed thrice with PBS on a rocker and stained with NucBlue^TM^ Fixed Cell ReadyProbes^TM^ Reagent (DAPI) (Invitrogen, Waltham, MA, USA) for 20 min at room temperature. The cells were washed thrice with PBS on a rocker (room temperature) and imaged with the EVOS M5000 fluorescence microscope (ThermoFisher Scientific, Sydney, NSW, Australia).

### 2.7. MPO ELISA

MPO ELISA was performed as per manufacturer’s instructions (Proteintech, Australia). Briefly, ECFCs were lysed in supplemented ice-cold RIPA lysis buffer and BCA assay was performed. Cell lysates were loaded in duplicates and incubated (2 h, 37 °C) and afterwards, the cells were washed with wash buffer. Anti-MPO antibody was added (1 h, 37 °C) after which the cells were washed and 2° antibody was added (40 min, 37 °C). The cells were washed again and 100 μL TMB substrate was added (20 min, 37 °C). The reaction was stopped with the addition of 100 μL of H_2_SO_4_ stop solution, and absorbance was measured (450 nm, correction wavelength: 630 nm) using a microplate reader (FLUOstar^®^ Omega, BMG Labtech, Ortenberg, Germany). Results were presented as MPO protein concentration:

(1) $$MPO\;protein\;concentration=\frac{MPO\;protein\;(pg)\;}{\;Cell\;lysate\;(\mu g)}$$

MPO protein concentration was determined by normalising ELISA results to the MPO standard curve which was then normalised to protein concentration. PBMCs were used as positive control.

### 2.8. RNA Extraction and qRT-PCR

RNA was extracted as per manufacturer’s protocol (Qiagen) and reverse transcription of the RNA was performed with the SuperScript IV VILO kit (Thermo Fisher Scientific, USA) with standard protocol. ECFC cDNA library was created and stored at −20 °C for gene expression analysis. Gene expression was analysed with SYBRGreen protocol (Thermo Fisher Scientific, USA) on the QuantStudio 12K Flex Real-Time PCR System. Primers for gene of interest were found from papers and checked for alignment to the human genome with the BLAST program. Supplementary Table S1 shows primers used in the experiment. The reaction was set up in 384-well plates with 10 μL of the reaction master mix. Triplicates and appropriate controls were performed for each gene and relative gene expression was analysed using the ΔΔCT method [25]. CT values > 35 were excluded/considered as having no expression for analysis [26].

### 2.9. MPO Activity Assay

The MPO activity assay was performed and calculated as per manufacturer’s instructions (Cat no: MAK068, Sigma-Aldrich, USA). Briefly, samples confirmed to have MPO protein via ELISA were selected and loaded onto 96-well clear plates. Reaction mixes containing MPO substrate (samples and positive control) or water (negative control) were made up and 50 μL was added to each positive control, sample and sample blank wells. Plates were incubated at room temperature for 30 min. A 2 μL volume of stop solution was added and mixed and allowed to incubate for 10 min at room temperature. A 50 μL volume of TNB reagent was subsequently added. The absorbance was read afterwards at 412 nm. MPO activity was compared to the TNB standard curve and calculated as per the following equation:

ΔA_412_ = A_412(sample blank)_ − A_412(sample)_

### 2.10. Flow Cytometry

Briefly, cells were washed with warm PBS, removed with trypsin and centrifuged at 700× *g* for 5 min. The supernatant was removed and cells were then re-suspended in fluorescent staining buffer. Cells were triple-stained and incubated with 5 μM MitoSOX Red, 20 nM MitoTracker Green and 50 nM DilC (1)5 (Invitrogen, USA) for 25 min at 37 °C. Cells were then centrifuged at 700× *g* for 5 min and re-suspended in Flurobrite DMEM. Cells were placed in the dark on ice until analysed using the LSRFortessa flow cytometer (BD Science). MitoSOX Red, MitoTracker Green and DilC (1)5 were excited with 561 nm, 488 nm and 640 nm lasers with emission filters of 585 nm, 530 nm and 670 nm used, respectively. Single-stain and no-stain controls were also added for compensation. For all samples, a minimum of 10,000 events were acquired. Data was exported to FlowJo (FlowJo, LLC, Ashland, OR, USA) for analysis (Supplementary Figure S1). Functional mitochondria was determined as ECFCs with both MitoSOX Red^low^ and MitoTracker Green^low^ populations.

### 2.11. Statistical Analyses

Data are expressed as the mean ± SEM for the indicated number of experiments. For statistical analysis between two groups, normality and variance tests were performed prior to selecting the appropriate *t*-test. Categorical variables are presented as frequencies and percentages, while continuous variables are reported as means ± standard deviations for normally distributed data, and medians with interquartile ranges for non-normally distributed data. To compare categorical variables, Pearson’s chi-squared test was utilised, while Student’s *t*-tests were employed for continuous variables. *p* values ≤ 0.05 were considered statistically significant. All statistical analyses were performed using GraphPad Prism (version 10.0.0., San Diego, CA, USA) and Jamovi (version 2.3., Sydney, NSW, Australia).

## 3. Results

The clinical and demographic features of patients from the 93 BioHEART-CT cohort with ECFCs used in this study are presented in Table 1. A total of 58 of the 93 patients (62.4%) available with ECFCs had CAD defined by CCTA criteria, while the remainder had no evidence of coronary atherosclerosis. On average, patients with CAD were 15 years older (*p* < 0.001) than non-CAD patients and were more likely to have hypertension (10.2% higher than non-CAD patients, *p* = 0.05) and statin use (33.2% higher than non-CAD, *p* = 0.002). This is consistent with the overall clinical breakdown of participants in the broader BioHEART-CT study [18].

### 3.1. MPO Protein Is Expressed and Co-Localised to the Membrane and Chromatin of ECFCs

**Figure 1:**
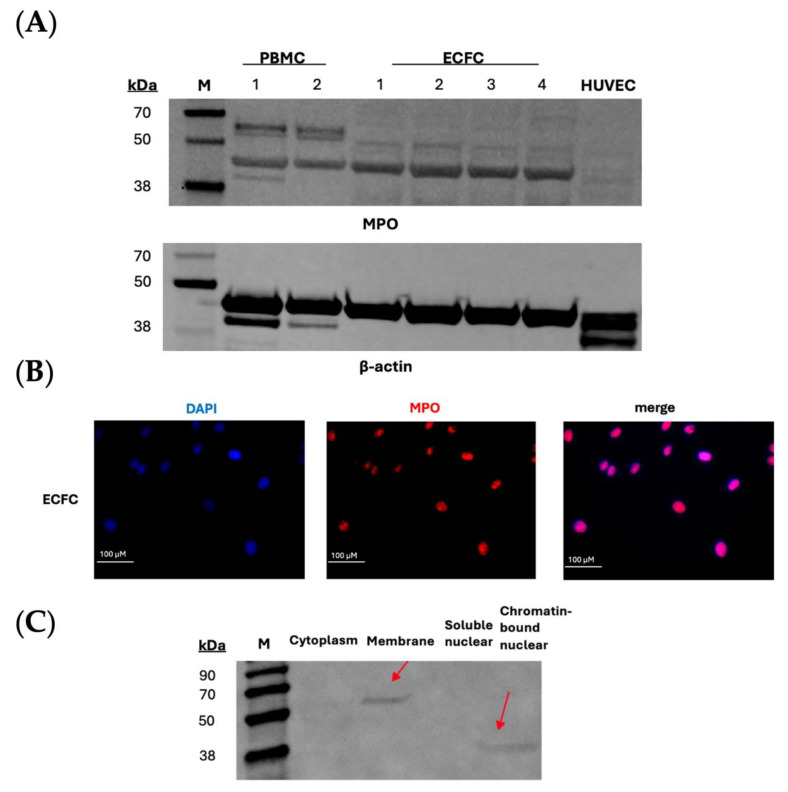
MPO protein expression in patient-derived ECFCs. (**A**) Representative Western blot of MPO protein expression in corresponding patient-derived ECFCs using anti-MPO antibody and anti-β-actin for loading control. (**B**) MPO protein is co-localised to the nuclei of patient-derived ECFCs. Representative immunocytochemistry images of MPO protein expression co-localised to the nucleus of patient-derived ECFCs, showing nuclei (blue) and MPO granules (red) within the cell (20× magnification). The scale bar represents 100 μM. (**C**) Representative Western blot of subcellular expression of MPO in patient-derived ECFCs at the soluble nuclear and chromatin-bound nuclear subfractions. At least three biological replicates were used. Original images can be found in Supplementary Materials file.

To establish a baseline and determine whether endothelial cells expressed MPO in comparison to immune cells, we performed Western blot analysis (Figure 1A). Autocatalytic MPO fragments with molecular masses of 43–47 kDa were observed in ECFCs [27,28]. Interestingly, some ECFC samples did not show any expression (Supplementary Figure S2). To further profile the patient cell lines, we performed ELISA assays to determine whether MPO was expressed in all ECFCs. Interestingly, our studies found that in the 41 patient ECFCs tested, MPO was detected in 29 samples (70.7%). However, there was no association with MPO protein expression and SMuRFs or CAD status, nor were there differences in intracellular MPO expression and activity between CAD and non-CAD patients (Supplementary File S1 and Supplementary Figure S3, respectively). This is interesting, as MPO is one of the most abundant enzymes within immune cells and it is usually released by them to cause an atherogenic effect.

Next, we investigated the co-localisation of MPO within MPO-expressing ECFCs. Immunocytochemistry studies initially revealed that MPO was co-localised to the nuclear region of ECFCs (Figure 1B). However, upon further investigation by Western blot analysis of subcellular protein fractionation, autocatalytic MPO fragments were identified in the chromatin-bound nuclear as well as heavy-chain MPO in the membrane extract of the ECFCs (Figure 1C). This finding is notable because MPO is typically located in the cytosol of immune cells. In addition, MPO enzyme was also investigated within the supernatant of ECFCs in vitro and, interestingly, 1 out of 41 samples (2.4%) had MPO (Supplementary Figure S4). This suggests that, in ECFCs, MPO does not undergo degranulation as it does in immune cells, indicating a secondary role for MPO in these cells.

### 3.2. MPO Gene Expression Is Inversely Associated with CAD, Particularly in Males

**Figure 2:**
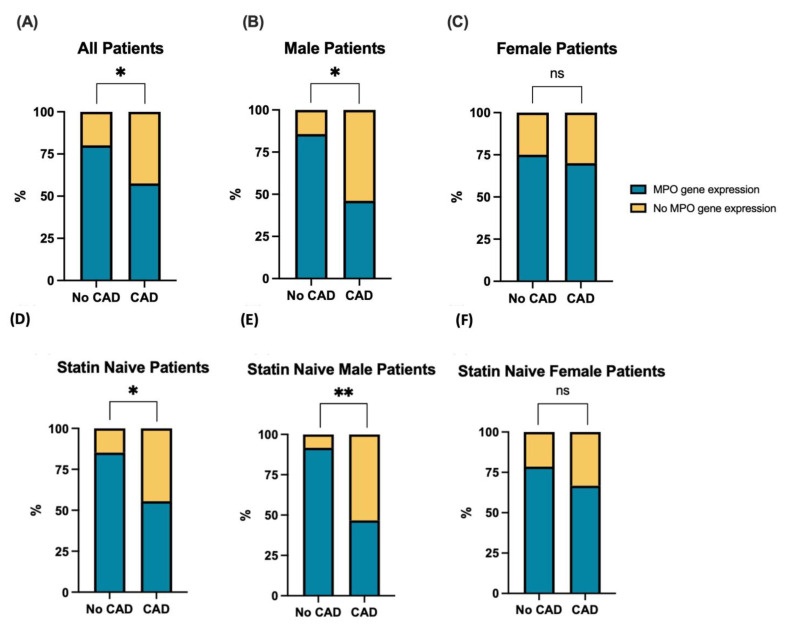
CAD patients were less likely to express MPO gene, as identified by qRT-PCR. Stacked bar plots showing the association between proportion in MPO gene expression and the presence of CAD in (**A**) all patients (*n* = 77; No CAD = 30, CAD = 47), (**B**) male patients (*n* = 40; No CAD = 14, CAD = 26) and (**C**) female patients (*n* = 36; No CAD = 16, CAD = 20). (**D**) Statin-naïve patients (*n* = 54; No CAD = 27, CAD = 27), (**E**) statin-naïve male patients (*n* = 27; No CAD = 12, CAD = 15) and (**F**) statin-naïve female patients (*n* = 26; No CAD = 14, CAD = 12). Statistical association was analysed using Pearson’s chi-square test (categorial variables). Categorical measurements are shown as percentages. * *p* < 0.05, ** *p* < 0.01.

We next investigated MPO mRNA expression. Across 77 patient-derived ECFCs, 66.23% had detectable MPO mRNA expression (CT values < 35) by qRT-PCR. There was no association between MPO mRNA expression and the presence of any SMuRFs (Supplementary File S2). We subsequently examined the potential association with a participant’s CAD status (Figure 2). We found that patients with CAD were three times less likely to express the MPO gene compared with non-CAD patients (odds ratio = 0.34, χ^2^ = 4.16, *p* = 0.04) (Figure 2A). The relationship between detectable MPO expression versus the presence or absence of CAD was more prominent in males. Male patients with CAD were seven times less likely to express the MPO gene as compared to non-CAD male patients (odds ratio = 0.14, χ^2^ = 5.93 *p* = 0.02; *n* = 40) (Figure 2B). However, no association was found within the female cohort (χ^2^ = 0.11, *p* = 0.74; *n* = 36) (Figure 2C). We next investigated whether there was differential mRNA expression of MPO between CAD and no CAD; no significant difference was found, including in male- and female-specific cohorts (Supplementary File S3).

Given the substantial anti-inflammatory benefit of statins in CAD, we evaluated the impact statin usage had on MPO expression. When taking only statin-naïve CAD individuals, there was no significant association between CAD and MPO protein expression (Supplementary Table S2). However, there was an exacerbated association between CAD and MPO gene expression, where those with CAD were 4.6 times less likely to express the MPO gene (odds ratio = 0.22, χ^2^ = 5.68, *p* = 0.02; *n* = 54) (Figure 2D). Within the male, statin-naïve cohort, the association was also more pronounced, in which those with CAD were 12.6 times less likely to express the MPO gene (odds ratio = 0.08, χ^2^ = 6.08, *p* < 0.01; *n* = 27) (Figure 2E). No association was found within the female sub-cohort after excluding those taking statins (χ^2^ = 0.47, *p* = 0.50; *n* = 26) (Figure 2F).

Having observed a relationship between the presence and absence of CAD and the detection of MPO expression, particularly in males, we examined the differences of relative fold gene expression with coronary disease in statin-naïve cohorts. No differences were observed in relative gene expression and CAD across the all-patient, male and female cohorts (Supplementary Figure S5).

Given the unusual location of MPO on immunohistochemistry (nuclear) and MPO gene expression being associated with CAD, we were also interested in investigating whether differences in MPO gene expression may have reflected different levels of differentiation of the ECFCs. Interestingly, no significant differences were found with MPO gene expression (yes/no) and the average days to spontaneous growth recorded during the ECFC-selective culture (Supplementary File S4).

### 3.3. MPO Gene Expression Is Associated with Dysregulated Mitochondrial Function

**Figure 3:**
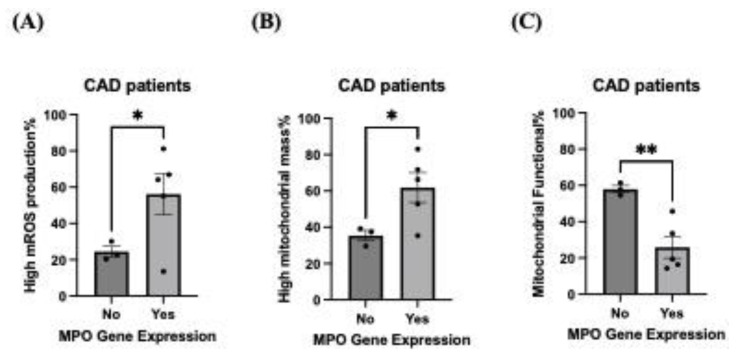
MPO gene expression is associated with dysregulated mitochondrial function and dynamics. (**A**–**C**) CAD ECFCs with MPO gene expression at baseline had increased (**A**) mROS production and (**B**) mitochondrial mass and decreased (**C**) mitochondrial function. N = 8; no MPO gene expression: N = 3, MPO gene expression: N = 5. * p < 0.05, ** p < 0.01. Data are represented as mean ± S.E.M. Welch’s *t*-test was performed.

Previously, it was found that patient-derived ECFCs had increased mitochondrial dysfunction (specifically, high mROS production) associated with CAD [18,19]. We wanted to investigate in ECFCs whether having MPO gene expression worsened mitochondria function. Interestingly, we found that when MPO was expressed in CAD ECFCs (*n* = 8, MPO gene expression = 5, no MPO gene expression = 3), it increased mROS production% (mean increase: 31.65 ± 11.81%, CI: 0.24% to 63.07%, *p* < 0.05) and mitochondrial mass% (mean increase: 26.44 ± 8.72%, CI: 3.95% to 48.93%, *p* < 0.05) whilst decreasing functional mitochondria% (mean decrease: 32.01 ± 6.31%, CI: −48.41% to −15.60%, *p* < 0.01) (Figure 3A–C). No difference was found in the non-CAD group (Supplementary Figure S6).

### 3.4. MPO Gene Expression Is Associated with Decreased Mitochondrial-Antioxidant Capacity

**Figure 4:**
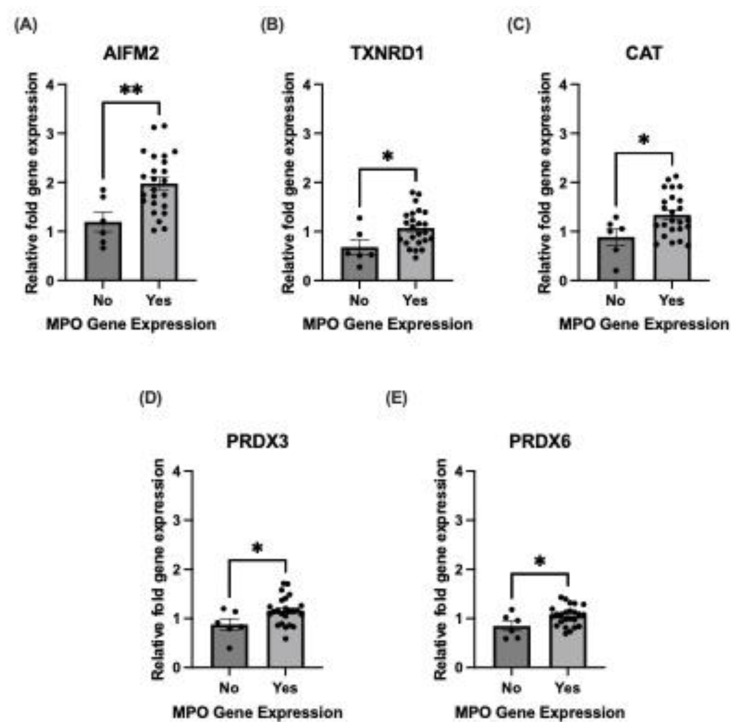
Antioxidant genes were upregulated with MPO gene expression in non-CAD patients. Relative fold gene expression was evaluated in ECFCs without CAD at baseline in (**A**) *AIFM2*, (**B**) *TXNRD1*, (**C**) *CAT*, (**D**) *PRDX3* and (**E**) *PRDX6*. Each sample was performed in triplicate. N = 30; no MPO gene expression: N = 6, MPO gene expression: N = 24. Data are represented as mean ± S.E.M. * *p* < 0.05, ** *p* < 0.01, Student’s *t*-test was performed.

Subsequently, we investigated how MPO gene expression affects antioxidant pathways. We obtained a gene set from a previously obtained ECFC transcriptome. Within the non-CAD cohort (*n* = 30; No CAD = 6, CAD = 24), we found that the mitochondrial-antioxidant genes *AIFM2* (mean fold change difference: 0.78 ± 0.27, CI: 0.24 to 1.33, *p* < 0.01), *TXNRD1* (mean fold change difference: 0.39 ± 0.17, CI: 0.05 to 0.73, *p* < 0.05), *CAT* (mean fold change difference: 0.46 ± 0.20, CI: 0.05 to 0.86, *p* < 0.05), *PRDX3* (mean fold change difference: 0.29 ± 0.13, CI: 0.02 to 0.55, *p* < 0.05) and *PRDX6* (mean fold change difference: 0.21 ± 0.10, CI: 0.01 to 0.40, *p* < 0.05) were significantly upregulated when MPO was expressed (Figure 4A–E). However, when we look within the CAD-cohort, these differences were nullified (Supplementary Figure S7).

## 4. Discussion

Whilst MPO has been implicated in exacerbating CAD and atherosclerosis more broadly, its expression and function in endothelial cells has been debated. Here, we are the first to report the intracellular presence of MPO within patient-derived ECFCs. In addition, we report that MPO has a potential transcriptional role associated with mitochondrial health and antioxidants.

The notion of endogenous MPO expression in endothelial cells is not entirely novel; however, the finding that it is differentially expressed based on a patient’s CAD status is interesting [14,15,18]. We have previously reported on the phenotypic imprint retained by ECFCs reflecting the CAD status of the individual, most distinctly with dysregulated mitochondrial redox signalling [18,19]. Previously, it was reported that ECFCs secrete low levels of MPO; however, it was not previously linked to specific gene or protein expression levels or to clinical status [29]. We also report something similar, with 2.4% of samples having observed MPO within the supernatant (Supplementary File S5). In endocardial endothelial cells of patients with chronic heart failure, it was also reported that only through noxious stimuli do they generate MPO [14]. Our study suggests otherwise, demonstrating that, in the absence of external stimuli, ECFCs can express endogenous MPO at both the gene and protein level (Figure 1 and Figure 2).

MPOs are best known to function within neutrophils and monocytes, where it is released through a process of degranulation to facilitate anti-microbial activity through the generation of hypochlorous acid (HOCl^−^) [5,8]. In these cells, MPO is classically located in the primary (azurophilic) granules [30]. This study suggests an alternative role is likely in ECFCs, with a potential protective role where MPO was observed in the chromatin-bound nuclear extract (Figure 1B,C). A paper by Murao et al. found that intra-nuclear MPO in human myeloid cells was able to protect DNA from strand scission-damage caused by reactive oxygen species (ROS) [31]. As such, this suggests a possible protective role in ECFCs, but further investigation is required. Furthermore, there was a higher presence of MPO gene in non-CAD patients, particularly pronounced in male cohorts and those who are statin-naïve (Figure 2). Exclusion of patients using statin usage was based on previous evidence indicating strong suppression of MPO gene expression in bone-marrow precursors [32,33], which ECFCs are suggested to originate from [34]. The results suggests that statin usage reduces the need for endogenous MPO protection in these cells. Moreover, when we explore the associations between redox-protective enzymes (Figure 4), it is fascinating that, when MPO is expressed within non-CAD patients, the mitochondrial-antioxidant genes (*AIFM2*, *TXNRD1*, *CAT*, *PRDX3* and *PRDX6*) are significantly upregulated. This seems to suggest a positive transcriptional regulatory function of MPO.

Whilst MPO has been associated with an antioxidative role, MPO in this paper has also been found to be detrimental to mitochondrial health. Here, we show that CAD patients that had MPO gene expression, compared to their counterparts (no MPO gene expression), exhibit increased mROS production and mitochondrial mass and decreased mitochondrial function (Figure 3A–C). This is synonymous with the canonical idea that MPO is detrimental to the progression of CAD and atherosclerosis [11,12]. A possible explanation is that ECFCs express exclusively autocatalytic MPO within the chromatin-bound nuclear extract (Figure 1C), which amplifies oxidative stress as well as creating a self-sustaining cycle of DNA damage which could have downstream effects on cellular components like the mitochondria [35].

In addition, the findings of male-favoured sex-specific differences of MPO in CAD contradict previously reported observations [4]. In the literature, MPO has been associated with cardiovascular events in women [4,36,37,38]. An explanation for the sex-specific difference could be due to higher MPO levels found in males compared to females in different diseases. Two studies found that males with cardiovascular disease had increased MPO plasma levels compared to their female counterparts [39,40]. A separate study also found that males with Parkinson’s disease had increased MPO levels within the cerebral spinal fluid (CSF) compared to females [41]. This suggests that a differential immune or inflammatory response exists between males and females along with other genetic and environmental factors. Further studies including genetic testing would be required to fully elucidate this. Considering the conflicting role of MPO in atherosclerosis, perhaps there are more unknown roles and functions of MPO that we are yet to unravel.

### 4.1. Limitations

It is worth noting that, as a preclinical model of CAD, our study suffers from several limitations. Previously, it was reported that MPO levels were elevated in the leucocytes and blood of patients with CAD [3]. However, translating our findings into clinical prognostic or therapeutic markers for CAD faces significant challenges. The rarity of ECFC growth [17,18,19,42,43], combined with the sporadic nature of MPO gene and protein expression in patient-derived ECFCs (Figure 1 and Figure 2), poses substantial limitations, underscoring the need for further research to address these challenges in pursuit of clinical translatability. Due to improper long-term storage conditions, some samples (RNA, protein, cellular) experienced significant degradation, resulting in poor overlap across different sample types (26.88%) and limited downstream analysis. Consequently, alternative samples were used to increase the sample size when necessary. Another limitation of the study is selection bias, as only patients with clinical indications for CCTA were included [21]. Additionally, the geographical isolation of Australia adds to the study’s constraints. These issues need to be addressed and validated through multi-national studies [21]. Another limitation of this study is the existence of polymorphic variants in the MPO gene, such as the MPO 463G/A polymorphism, which has been associated with an increased risk of CAD and heightened oxidative stress [44]. This polymorphism could potentially explain the elevated oxidative stress observed in CAD patients with MPO gene expression in our study. However, we did not perform pharmacogenetic testing to screen for MPO polymorphisms in our patient cohort, which may have provided further insights into the variability in oxidative stress responses. Due to the rarity of patient-derived ECFCs, we were limited in our ability to conduct extensive polymorphism screening. Future studies should consider including pharmacogenetic analysis to better elucidate the role of MPO polymorphisms in oxidative stress and drug sensitivity in CAD patients.

### 4.2. Future Directions

Future studies should investigate MPO expression and activity in the endothelium near or at coronary lesions. In addition, polymorphisms in the promoter of the MPO gene and coding regions have been reported to be associated with cardiovascular disease [44,45]. It would be important to assess in the MPO-expressing ECFCs what polymorphisms are being exhibited. Lastly, given MPO’s interplay between cardiovascular and immune systems, in vivo studies to assess MPO activity and function in atherosclerotic endothelium would be beneficial to understanding the dual role of MPO.

## 5. Conclusions

MPO serves as a valuable marker for understanding oxidative stress in a range of clinical settings. Elevated MPO levels within the patient serum have been linked to an increased risk of cardiovascular diseases, including heart attacks and atherosclerosis.

For the first time, our study identified patient-derived ECFCs expressing the MPO gene and protein. Interestingly, individuals with a history of CAD exhibited lower MPO gene expression, observed in statin-naïve cohorts. Male CAD patients with clinically actionable CAD displayed significantly lower MPO gene expression, suggesting a strong correlation with clinically actionable CAD. Additionally, low MPO protein levels were significantly associated with patients with severe CAD, implying a potential protective role of intracellular MPO in CAD patients’ endothelial cells. These findings collectively support the hypothesis that intracellular MPO may play a protective role in CAD patients’ endothelial cells.

## Figures and Tables

**Table 1:** Baseline characteristics of BioHEART-CT population. Associations between clinical characteristics and CAD.

Clinical Characteristics	Overall (*n* = 93)	CAD		*p*-Value
		Yes (*n* = 58)	No (*n* = 35)	
**Age, median (IQR)**	**62 (19.0)**	68 (17.0)	53 (10.3)	**<0.001**
Female, n (%)	43 (46.2)	24 (41.4)	19 (54.3)	0.23
Hypertension, n (%)	33 (35.5)	25 (43.1)	8 (22.9)	**0.05**
Diabetes, n (%)	5 (5.4)	4 (6.9)	1 (2.9)	0.40
Hypercholesteremia, n (%)	52 (55.9)	34 (58.6)	18 (51.4)	0.50
Current smoker, n (%)	7 (7.5)	6 (10.3)	1 (2.9)	0.39
Statins-n (%)	24 (30)	21 (42.9)	3 (9.7)	**0.002**

Statistical association was analysed using Student’s *t*-test (continuous variables) and Pearson’s chi-square test (categorial variables). Continuous measurements are shown as median with interquartile range (IQR; due to non-Gaussian distribution) and categorical variables as percentages. Bold refers to statistically significant result.

## Data Availability

The data presented in this study are available on request from the corresponding author.

## Supplementary Materials

The following supporting information can be downloaded at: https://www.mdpi.com/article/10.3390/biom14101308/s1, Supplementary File S1–S4; Supplementary File S5—MPO ELISA supernatant. Figure S1. (A) Flow gating strategy for ECFCs. (B) Representative flow cytometry images showing mitochondrial signatures from ECFCs of patients with and without CAD. MitoSOX and MitoTracker analyses were used to measure mROS production and mitochondrial area. Dysfunctional Mitochondria population% is derived as a combination of MitoSOX Red^high^ and MitoTracker Green^high^ gating. Functional Mitochondria population% is derived as 100% − Dysfunctional mitochondria population%. Figure S2. MPO protein expression in ECFCs. Figure S3. MPO activity between CAD and non-CAD. Figure S4. MPO presence in the supernatant of cultured ECFCs. Figure S5. Relative MPO fold gene change between CAD and non-CAD in all patients, males and females. Figure S6. No difference in mitochondrial function. Figure S7. Mitochondrial gene expression in the CAD-specific ECFC cohort that expressed/didn’t express MPO; Table S1: List of primers used for qRT-PCR. Table S2. Associations between MPO protein expression and CAD. Table S3. Associations between MPO gene expression and CAD. Original images can be found in Supplementary Materials file.
