# Supplementary material for: Expression of Myeloperoxidase in Patient-Derived Endothelial Colony-Forming Cells—Associations with Coronary Artery Disease and Mitochondrial Function
Source: Biomolecules. 2024 Oct 16;14(10):1308. doi: 10.3390/biom14101308 (PMC11505856; doi:10.3390/biom14101308)
Supplement: Supplementary file 1 [file biomolecules-14-01308-s001.zip › Supplementary tables and figures.pdf]

Tables

Supplementary Table S1: List of primers used for qRT-PCR

Gene	Forward sequence (5'-3')	Reverse sequence (5'-3')
MPO	CCGGGATGGTGATCGGTTTT	CAGATGATCCGGGGCAATGA
GAPDH	AGCCACATCGCTCAGACAC	GCCAATACGACCAAATCC

Supplementary Table S2. Associations between MPO protein expression and CAD

	MPO protein expression	Overall	CAD		*p*-value
			**Yes**	**No**	
All participants	(%)	(29.27)	(29.63)	(28.57)	0.94
	Statin (%)	(33.33)	(42.86)	(25.00)	0.46
Male	(%)	(21.43)	(33.33)	(0.00)	0.15
	Statin (%)	(28.57)	(50.00)	(0.00)	0.15
Female	(%)	(11.11)	(0.00)	(33.33)	0.13
	Statin (%)	N/A	N/A	N/A	N/A

Statistical association was analysed using Pearson's chi-square test (categorial variables). Categorical measurements are shown as percentages. Bold refers to statistically significant result.

Supplementary Table S3. Associations between MPO gene expression and CAD

	MPO gene expression	Overall	CAD		*p*-value
			**CACS=0**	**CACS>0**	
All participants	(%)	(66.23)	(80.00)	(57.45)	**0.04**
	Statin naïve (%)	(70.37)	(85.19)	(55.56)	**0.02**
Male	(%)	(60.00)	(85.71)	(46.15)	**0.02**
	Statin naïve (%)	(66.37)	(91.67)	(46.67)	**0.01**
Female	(%)	(72.22)	(75.00)	(70.00)	0.74
	Statin naïve (N, %)	19 (73.08)	11 (78.57)	8 (66.67)	0.50

Statistical association was analysed using Pearson's chi-square test (categorial variables). Categorical measurements are shown as percentages. Bold refers to statistically significant result.

Figures

Supplementary Figure S1. (A) Flow gating strategy for ECFCs. (B) Representative flow cytometry images showing mitochondrial signatures from ECFCs of patients with and without CAD. MitoSOX and MitoTracker analyses were used to measure mROS production and mitochondrial area. Dysfunctional Mitochondria population% is derived as a combination of MitoSOX Red^{high} and MitoTracker Green^{high} gating. Functional Mitochondria population% is derived as 100% - Dysfunctional mitochondria population%.

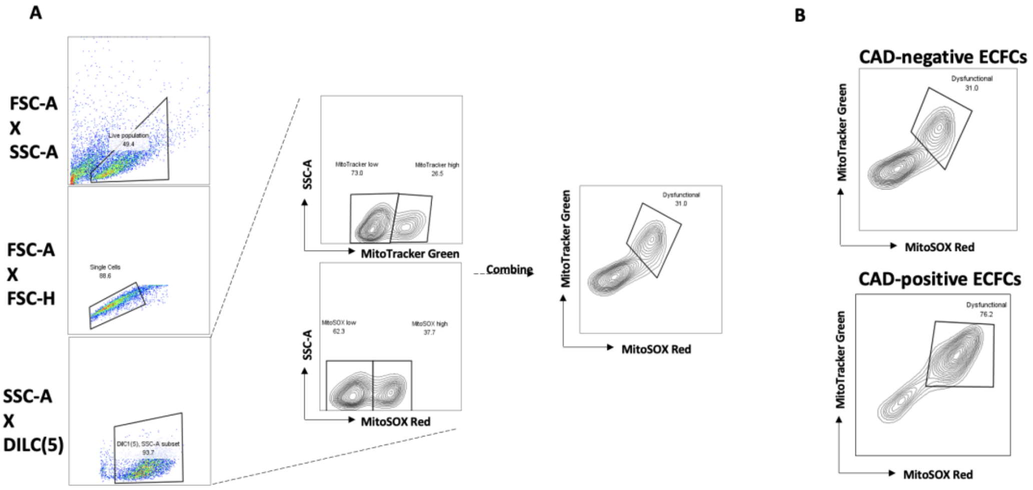

Supplementary Figure S2. MPO protein expression in ECFCs

Proportion Test (2 Outcomes)

Binomial Test

	Level	Count	Total	Proportion	p
MPO protein expression	No	29	41	0.70732	0.012
	Yes	12	41	0.29268	0.012

Note. H_a is proportion ≠ 0.5

Supplementary Figure S3. MPO activity between CAD and non-CAD

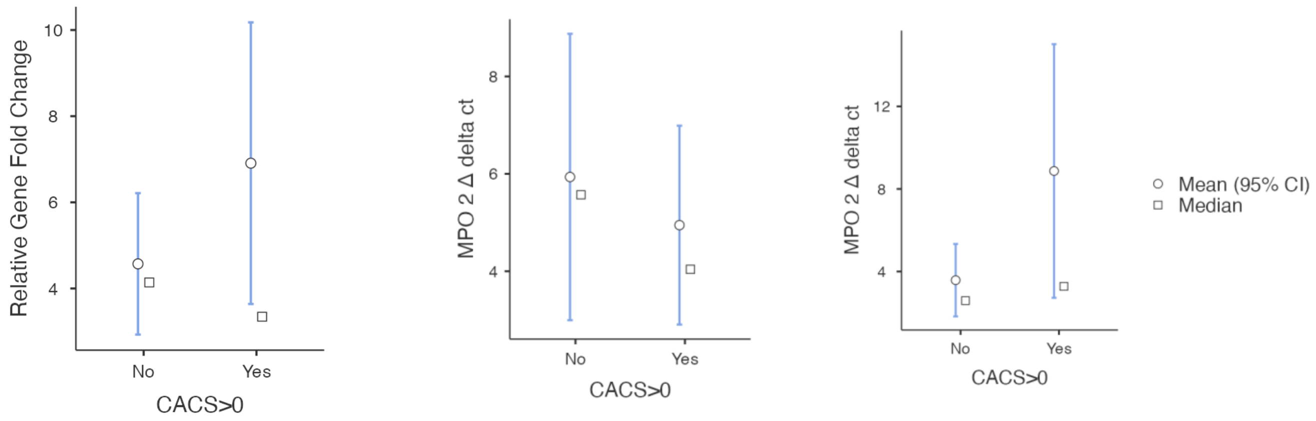

Supplementary Figure S4. MPO presence in the supernatant of cultured ECFCs

Proportion Test (2 Outcomes)

Binomial Test

	Level	Count	Total	Proportion	p
MPO protein expression in supernatant	Yes	1	42	0.02381	<.001
	No	41	42	0.97619	<.001

Note. H_a is proportion ≠ 0.5

Supplementary Figure S5. Relative MPO fold gene change between CAD and non-CAD in all patients, males and females. Non-parametric independent samples t-test between MPO mRNA expression level between CAD and no CAD in (A) all patients, (B) male patients and (C) female patients.

(A) (B)

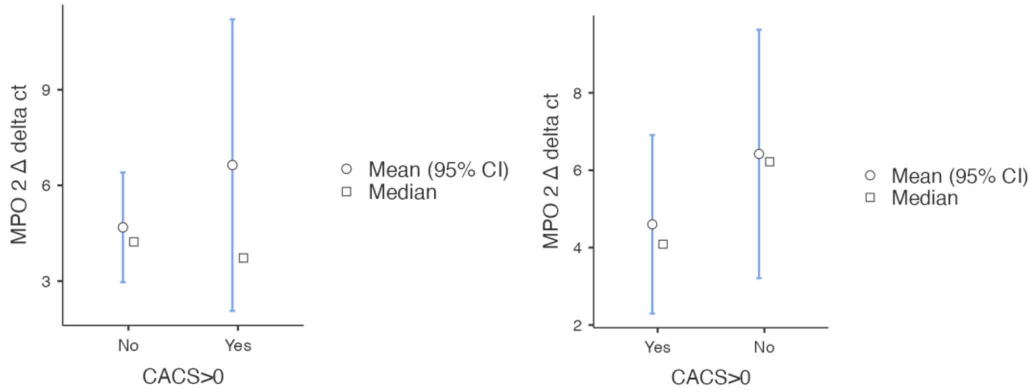

(C)

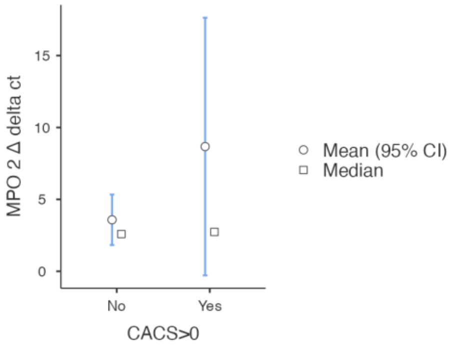

Supplementary Figure S6. No difference in mitochondrial function. Non-parametric independent samples t-test between MPO gene expression and different metrics of mitochondria function. A) Mitochondrial mass, B) mROS production and C) mitochondrial function.

(A)

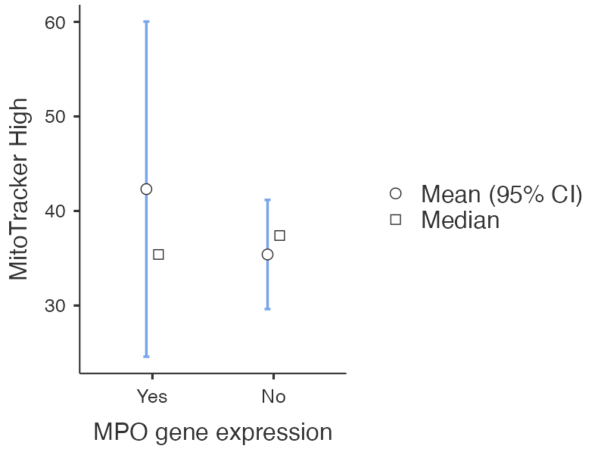

(B)

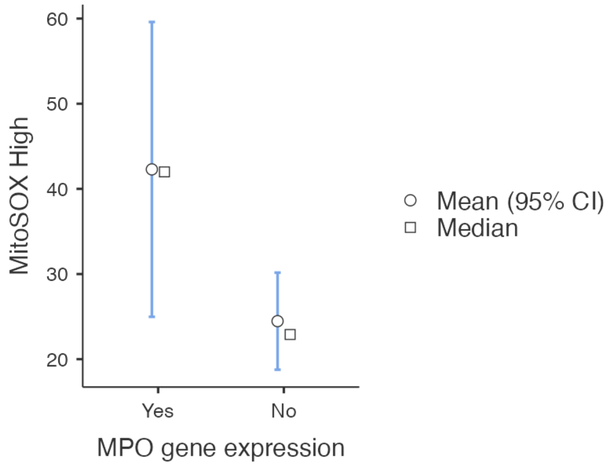

(C)

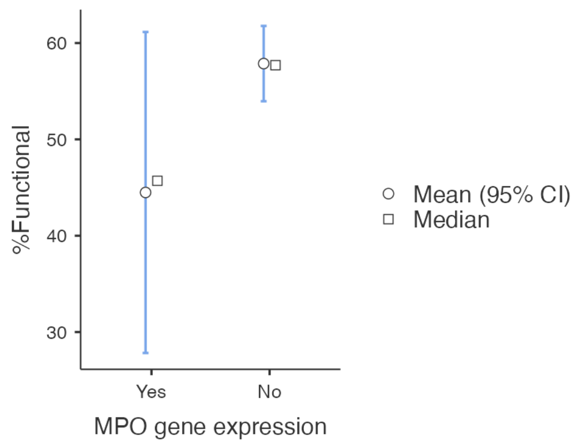

Supplementary Figure S7. Mitochondrial gene expression in the CAD-specific ECFC cohort that expressed/didn't express MPO. (A) AIFM2 (B) TXNRD1 (C) PRDX3 (D) CAT (E) PRDX6. Student's t-test was performed.

(A)

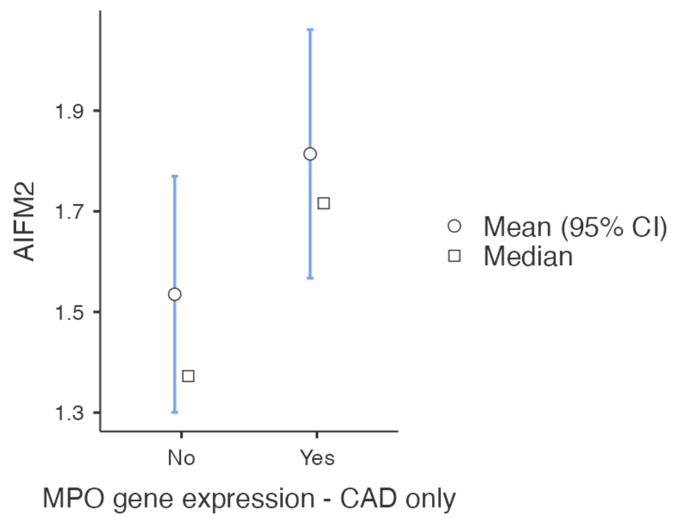

(B)

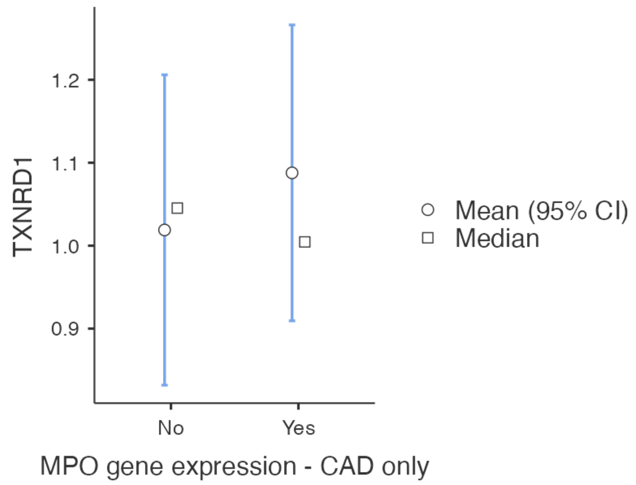

(C)

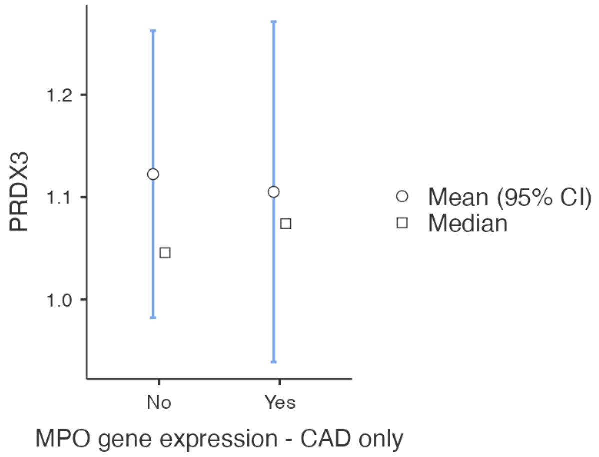
PRDX3
1.2
1.1
1.0
No
Yes
MPO gene expression - CAD only
Mean (95% CI)
Median

(D)

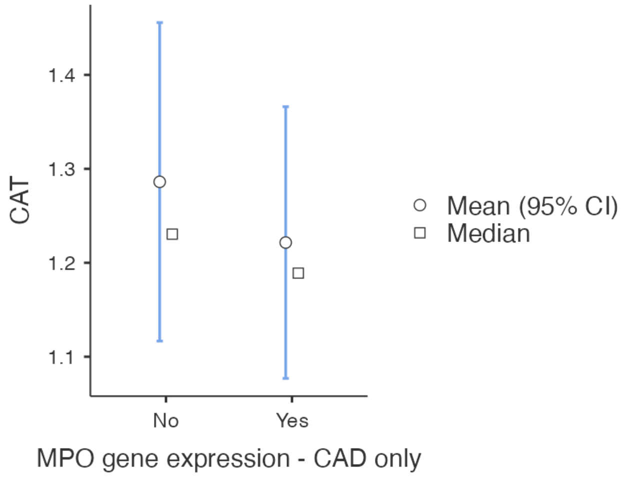
CAT
1.4
1.3
1.2
1.1
No
Yes
MPO gene expression - CAD only
Mean (95% CI)
Median

(E)

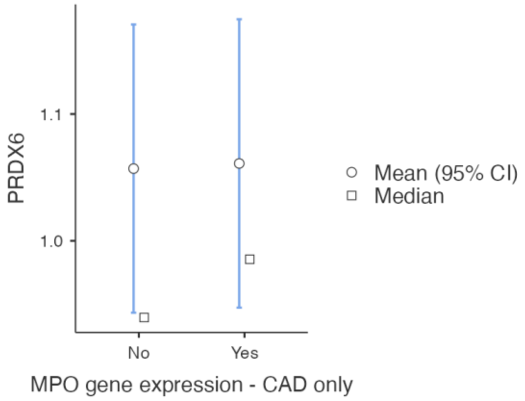
PRDX6
1.1
1.0
No
Yes
MPO gene expression - CAD only
Mean (95% CI)
Median
