# Supplementary figures and images for: Expression of Myeloperoxidase in Patient-Derived Endothelial Colony-Forming Cells—Associations with Coronary Artery Disease and Mitochondrial Function
Source: Biomolecules. 2024 Oct 16;14(10):1308. doi: 10.3390/biom14101308 (PMC11505856; doi:10.3390/biom14101308)

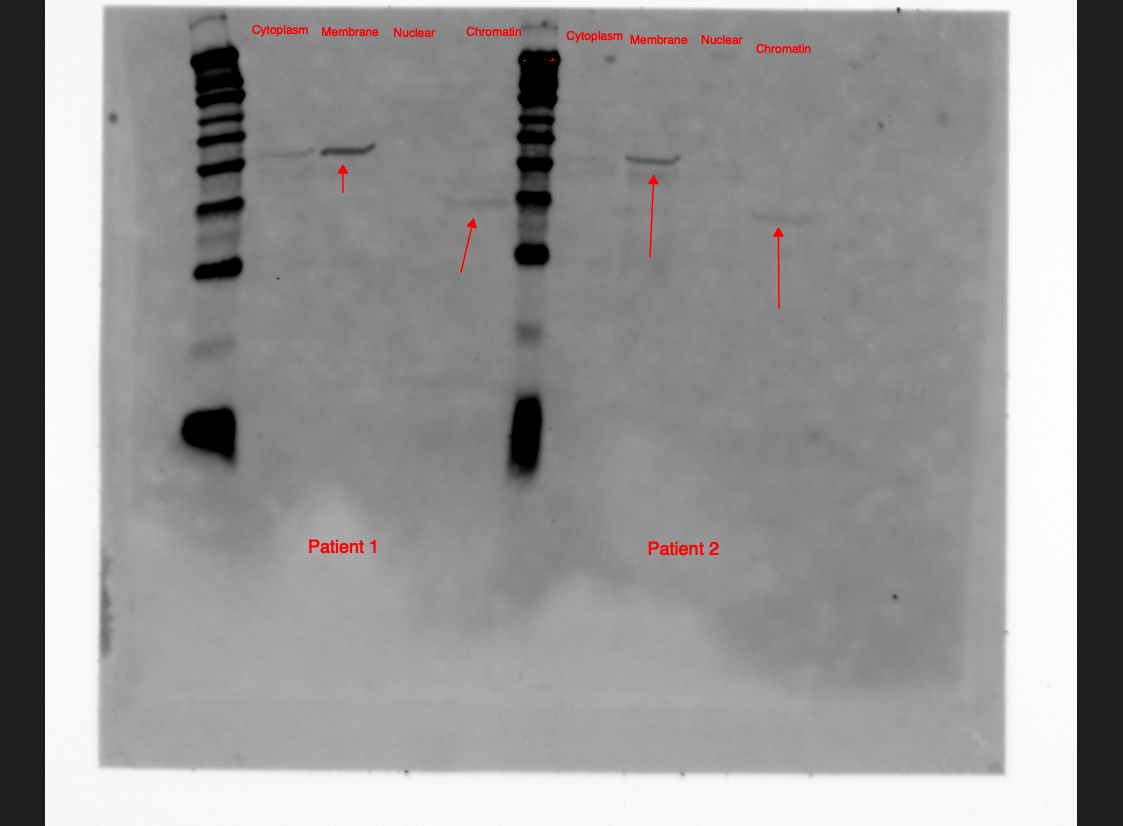

Supplement: Supplementary file 1 [file biomolecules-14-01308-s001.zip › Patient 1 and Patient 2 MPO subfractionation - Figure 1C.tif]

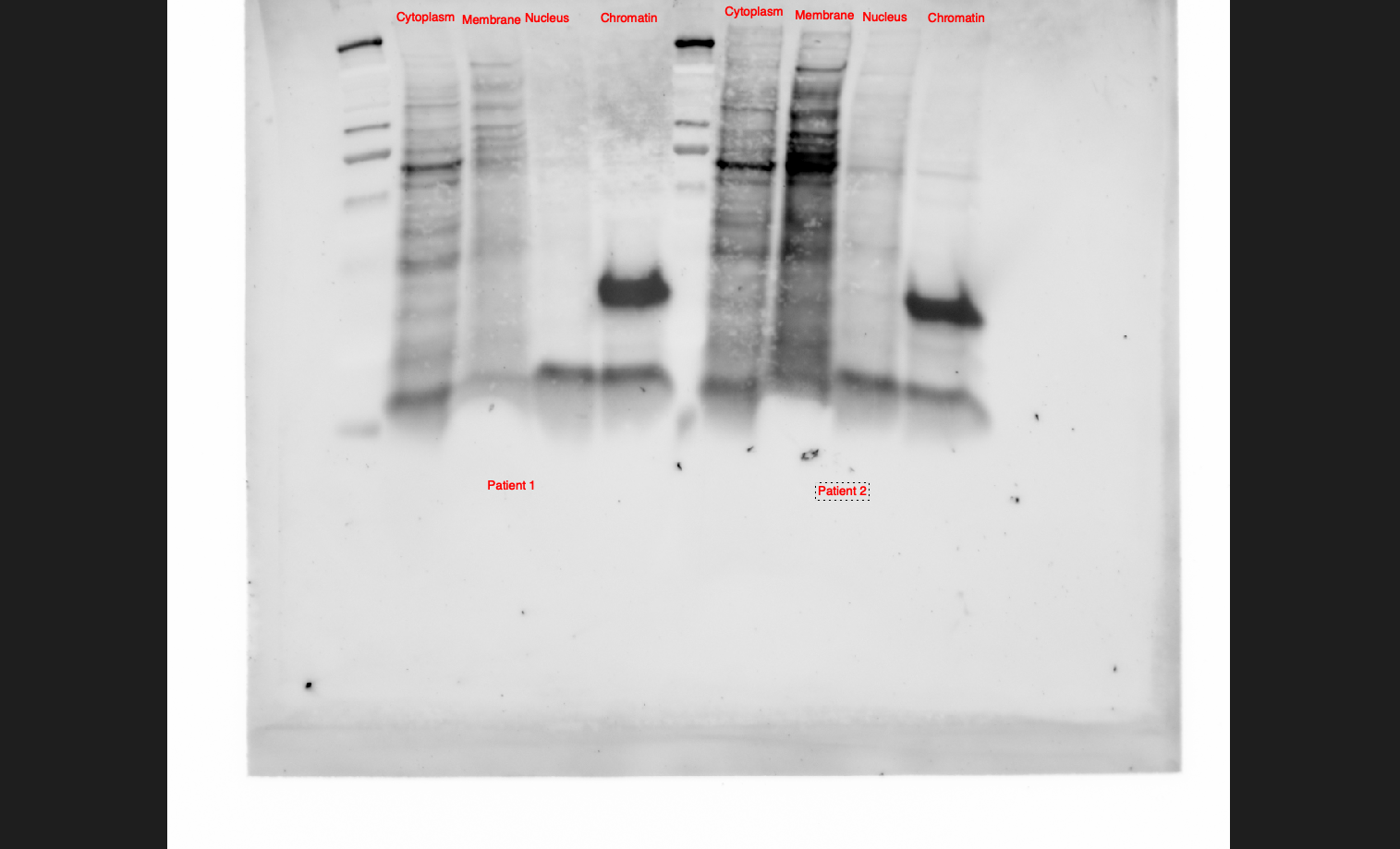

Supplement: Supplementary file 1 [file biomolecules-14-01308-s001.zip › Patient 1 and Patient MPO total protein stain.tif]

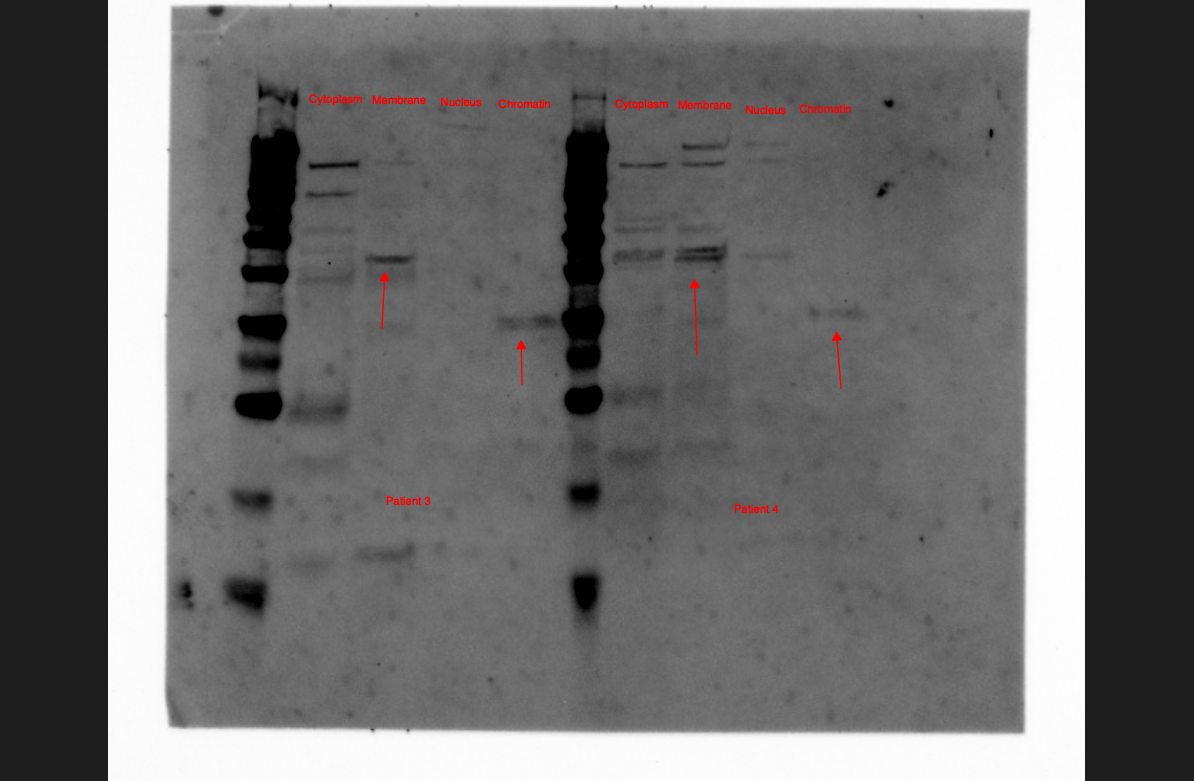

Supplement: Supplementary file 1 [file biomolecules-14-01308-s001.zip › Patient 3 and 4 subfractionation.tif]

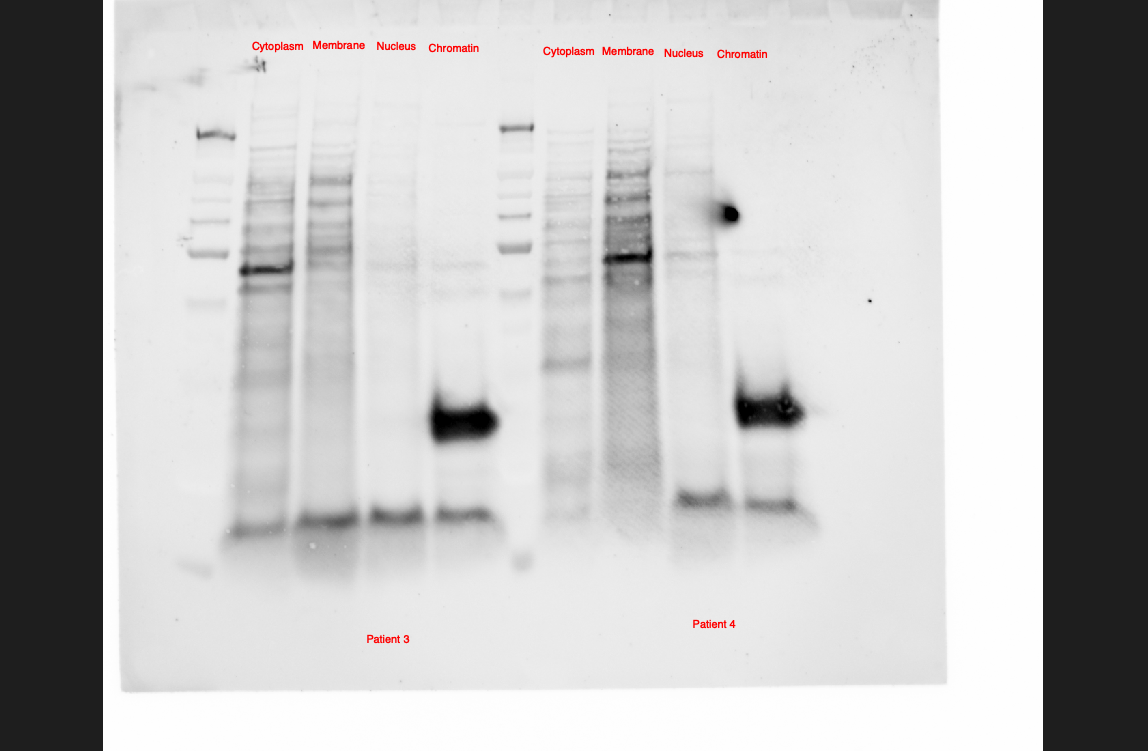

Supplement: Supplementary file 1 [file biomolecules-14-01308-s001.zip › Patient 3 and Patient 4 MPO total protein stain.tif]

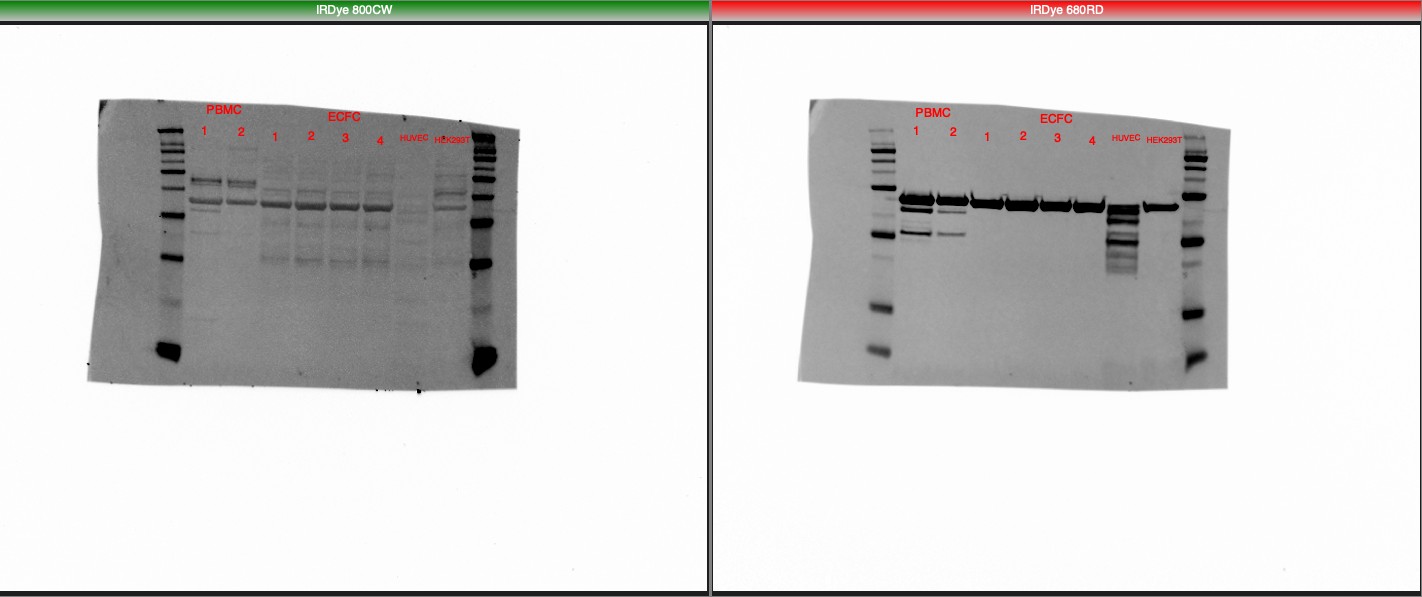

Supplement: Supplementary file 1 [file biomolecules-14-01308-s001.zip › Whole cell lysate western blot - MPO.tif]
